# iBrick: A New Standard for Iterative Assembly of Biological Parts with Homing Endonucleases

**DOI:** 10.1371/journal.pone.0110852

**Published:** 2014-10-20

**Authors:** Jia-Kun Liu, Wei-Hua Chen, Shuang-Xi Ren, Guo-Ping Zhao, Jin Wang

**Affiliations:** 1 CAS Key Laboratory of Synthetic Biology, Institute of Plant Physiology and Ecology, Shanghai Institutes for Biological Sciences, Chinese Academy of Sciences, Shanghai, China; 2 State Key Lab of Genetic Engineering & Center for Synthetic Biology, Department of Microbiology and Microbial Engineering, School of Life Sciences, Fudan University, Shanghai, China; 3 Shanghai-MOST Key Laboratory of Disease and Health Genomics, Chinese National Human Genome Center at Shanghai, Shanghai, China; 4 Department of Microbiology and Li Ka Shing Institute of Health Sciences, The Chinese University of Hong Kong, Prince of Wales Hospital, Shatin, New Territories, Hong Kong SAR, China; Imperial College London, United Kingdom

## Abstract

The BioBricks standard has made the construction of DNA modules easier, quicker and cheaper. So far, over 100 BioBricks assembly schemes have been developed and many of them, including the original standard of BBF RFC 10, are now widely used. However, because the restriction endonucleases employed by these standards usually recognize short DNA sequences that are widely spread among natural DNA sequences, and these recognition sites must be removed before the parts construction, there is much inconvenience in dealing with large-size DNA parts (*e.g*., more than couple kilobases in length) with the present standards. Here, we introduce a new standard, namely iBrick, which uses two homing endonucleases of I-SceI and PI-PspI. Because both enzymes recognize long DNA sequences (>18 bps), their sites are extremely rare in natural DNA sources, thus providing additional convenience, especially in handling large pieces of DNA fragments. Using the iBrick standard, the carotenoid biosynthetic cluster (>4 kb) was successfully assembled and the actinorhodin biosynthetic cluster (>20 kb) was easily cloned and heterologously expressed. In addition, a corresponding nomenclature system has been established for the iBrick standard.

## Introduction

Synthetic biology is a newly emerging discipline that learns from the engineering principles and uses complex combinations of DNA components to construct biological systems with new properties [1], [2]. The development of efficient methods for *de novo* synthesis of biological parts from a pool of oligos has greatly promoted the progress of synthetic biology. Nowadays, more and more companies are providing commercial services for *de novo* DNA synthesis, and most of them are using the polymerase chain assembly (PCA) [3]–[7] method to synthesize the designed DNA pieces up to several kilobases. Once small pieces are obtained, multiple approaches, *e.g.* BioBricks standard [8]–[10], Gibson assembly [11]–[13] and Master Ligation [14], are now available for assembling them into genes, pathways, modules or even a whole genome.

Among these standards (or techniques), BioBricks standard, which was originally introduced by Knight *et al.* (http://hdl.handle.net/1721.1/21168), is a milestone in the field of synthetic biology. A BioBricks part is a piece of DNA sequence flanked by XbaI and SpeI restriction sites on their 5′ and 3′ ends, respectively. Because XbaI and SpeI digested DNAs have compatible cohesive ends, they can be ligated head-to-tail to generate a hybrid DNA piece with a scar, and the resulted DNA piece is flanked by XbaI and SpeI again, which can be taken as a new BioBricks part for further iterative assembly. Techniques used in BioBricks assembly are merely DNA restriction/ligation and are easy to learn and to be widely adopted. On the other hand, once a BioBricks part is created and characterized, it can be reused in a variety of projects with different purposes. Built on the basis of BioBricks standard, the International Genetically Engineered Machine Competition (iGEM) has not only inspired young scientists in the field of synthetic biology but also promoted the sharing of biological resources.

However, the original BioBricks assembly scheme (BBF RFC 10, http://hdl.handle.net/1721.1/45138) produces an internal scar of 8 base pairs (bps) and has an in-frame stop codon. Therefore, it is not suitable for creation of fusion proteins, and certainly limits its landscape of potential applications. In fact, a series of improvements have been made to overcome the shortcomings of the original BioBricks standard thereafter. For example, BBF RFC 25 (http://hdl.handle.net/1721.1/45140) adds an NgoMIV site and an AgeI site in the prefix and suffix of RFC 10, respectively. As a consequence, BBF RFC 25 produces a scar of ACCGCC, encoding Thr-Gly, and supports the construction of plasmids for expression of in-frame fusion proteins. In addition, BglBricks [9], which is assigned BBF RFC 21 and uses BamHI and BglII instead of XbaI and SpeI, has addressed several key problems associated with the original BioBricks standard and is therefore a standard of great potential.

With the progress of synthetic biology, the demand of synthesis of long DNA sequences increases. Although the cost of DNA synthesis has significantly dropped in the past few years, *de novo* synthesis of large pieces of DNA sequences is still too expensive for most labs to afford, and it is also time-consuming. On the other hand, there exist numerous natural genetic materials, *e.g.* antibiotic biosynthetic gene clusters, many of which can actually be directly used for analysis without codon optimization or other manipulations. However, as these large pieces usually contain many recognition sites of type IIP and IIS restriction enzymes (REs) which excludes the direct usage of the present type II RE-based BioBricks standards [15]–[17]. Although internal restriction sites can be eliminated by synonymous base mutagenesis, it is practically unfeasible with regard to the cost of time and money. Moreover, base substitution may bring uncertainties in the phenotypes of the DNA sequences probably due to the influences of the synonymous single nucleotide polymorphisms (SNPs) [18]–[20]. Although other methods, such as PSA [21], Gibson Assembly and Master Ligation can be adopted, they do not allow convenient reuse of the constructed materials.

**Figure 1:**
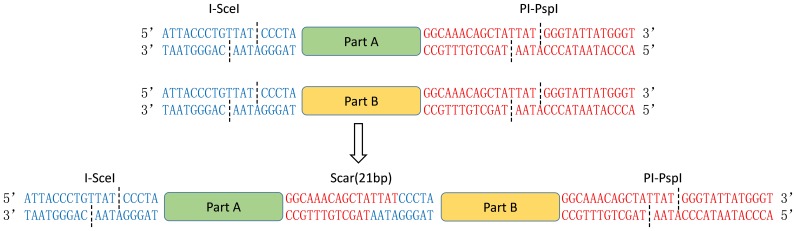
DNA sequences of the prefix, suffix and scar in iBrick assembly. Parts A and B are prefixed with I-SceI (shown in blue) and suffixed with PI-PspI (shown in red). After cleavage, compatible cohesive end (“TTAT”) can be ligated together to produce a 21-bp scar between parts. When translated in frame, this 21-bp DNA sequence encodes 7 amino acids.

**Figure 2:**
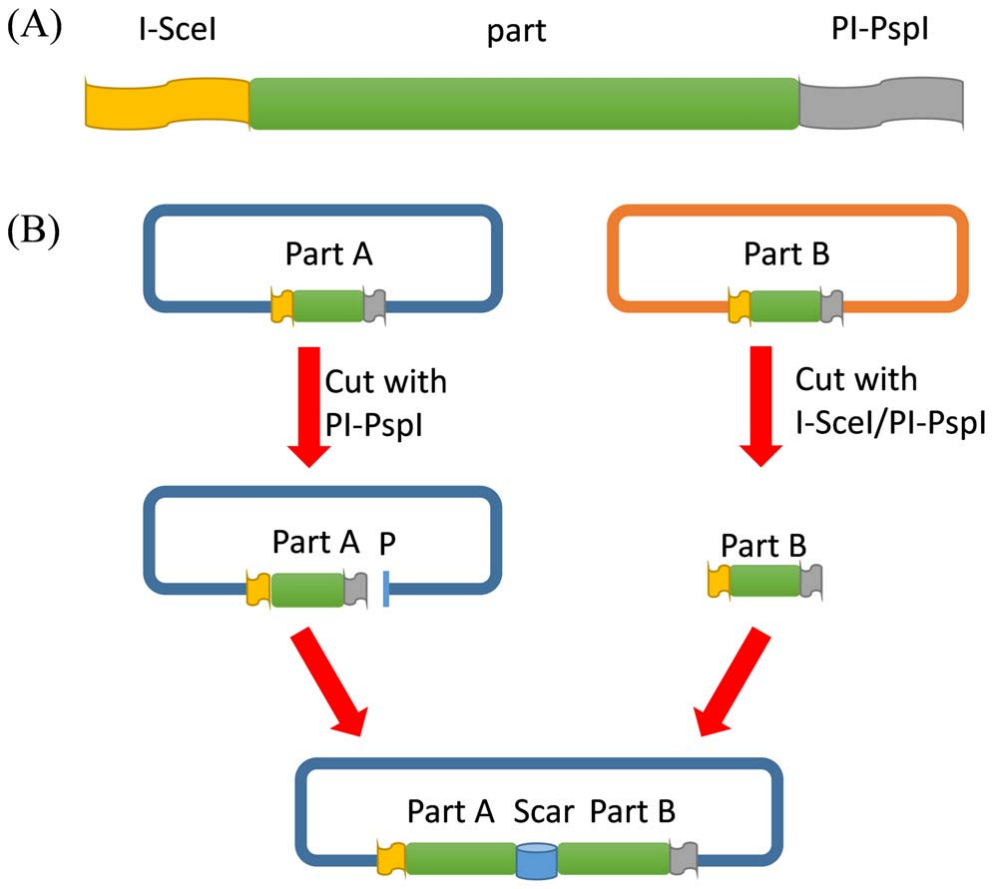
Schematic diagram of iBrick forward assembly. (A) Construction of iBrick parts, which should be prefixed and suffixed in iBrick standard. (B) Forward assembly procedure. Part B is firstly released with I-SceI and PI-PspI digestion, and is then inserted into the PI-PspI digested vector, obtaining an assembled part AB. Alternatively, the vector can also be cut with I-SceI followed by insertion of part B to obtain part BA, which is designated the reverse assembly.

Here, we introduce a new standard designated iBrick for iterative assembly of biological parts, which employs two homing endonucleases (HEs) of I-SceI [22]–[24] and PI-PspI [25], which produce compatible non-palindromic cohesive ends of “TTAT”. Parts in iBrick standard are prefixed with I-SceI and suffixed with PI-PspI (**Fig. 1**). As the cohesive ends produced *via* I-SceI and PI-PspI digestion are non-palindromic, the parts can be assembled in a defined order in iBrick standard (**Fig. 2**), similar to those using the BioBricks standards. In addition, because HEs used in iBrick recognize DNA sequences longer than 18 bps, which are much longer than that of the conventional type II REs and are thus extremely rare in natural DNA sequences, there is usually no need for modification of the DNA sequences regardless of their length. With the use of iBrick standard, we showcase here successful assembly of the carotenoid biosynthetic cluster (*crtEBI*) and convenient manipulation of the actinorhodin biosynthetic cluster (*act*) from *Streptomyces coelicolor* for heterologous expression in a thermophilic *Streptomyces* strain.

## Materials and Methods

### Bacterial strains, media, reagents and primers

*E. coli* strain DH5α was used for cloning of plasmids with pUC replication origin (pUCori); strain EPI300 (Epicentre) was used for cloning oriV-containing plasmids; and strain ET12567 was used for conjugation with *Streptomyces sp*. 4F [26]. *E. coli* strains were grown in Luria-Bertani medium [27], while *Streptomyces sp*. 4F was grown on mannitol soya flour [28] plates for spore preparation and on R2YE plates [29] for actinorhodin production.

Enzymes used in this study (unless specified) were purchased from NEB. GeneRuler 1-kb DNA ladder was purchased from Thermo Fermentas and λ-HindIII ladder was from TAKARA. High fidelity DNA polymerase KOD Plus Neo was purchased from TOYOBO. The lycopene standard was purchased from Sigma-Aldrich.

Primers used in this study were listed in **Table S1**.

### Protocol for construction of the iBrick plasmids

To *de novo* construct the base plasmids for iBrick assembly, beta-lactamase encoding gene [21], T4-g32 terminator, pUCori together with the *crtE* gene were used to construct the base plasmid pIB1A1_0-C000001. Specifically, *bla* was PCR amplified and the forward primer used contained the T4-g32 terminator sequence. The amplicon was then digested with NotI and phosphorylated with T4 polynucleotide kinase (PNK). Similary, pUCori was PCR amplified from pUC18 plasmid [30] and then the PCR product was cut with NotI at one end before it was ligated to *bla* to construct plasmid pJK5. Notably, several rare cutting enzymes that recognize 8-bp sites, *i.e.* PacI, NotI, SwaI and AscI, were used to link the replication origin, ampicillin resistance cassette and the terminator sequences in pJK5 (**Fig. S1**). Then, the *crtE* gene of the lycopene biosynthetic cluster was amplified from BioBrick BBa_K274200, flanked with SwaI/I-SceI sites at the 5′ end and PI-PspI/AscI sites at the 3′ end, and was then digested with SwaI and AscI and inserted into the same sites in pJK5 to obtain pIB1A1_0-C000001, in which two HE sites of I-SceI and PI-PspI were successfully introduced. Therefore, vector pIB1A1_0 prepared from I-SceI and PI-PspI double digestion of pIB1A1_0-C000001 was used as the base vector for creating small parts for iBrick assembly.

In addition to the ampicillin resistance cassette, other antibiotic resistance genes, including kanamycin, chloramphenicol and hygromycin were also amplified with the same flanking restriction sites of PacI and NotI, and were further used to substitute the *bla* gene in pIB1A1_0-C000001 to obtain base plasmids of pIB1K1_0-C000001, pIB1C1_0-C000001 and pIB1H1_0-C000001, respectively (**Fig. S4**).

The replication origin of pCC2FOS (Epicentre) containing both the F factor-based partitioning system and single-copy origin of ori2 and the inducible oriV [31] was PCR amplified and digested with NotI/SwaI before cloning into pIB1A1_0-C000001, obtaining pIB2A1_0-C000001. Similarly, the *bla* gene was replaced by *kan*, *apr* and *hyg* to obtain base plasmids of pIB2K1_0-C000001, pIB2Am1_0-C000001and pIB2H1_0-C000001, respectively (**Fig. S4**). Vectors prepared from these base plasmids were used as base vectors for cloning of large iBrick parts.

### Procedure of assembly and verification of the lycopene biosynthetic cluster (*crt*)

In addition to *crtE*, other lycopene biosynthesis genes, *i.e. crtB* and *crtI*, and the arabinose inducible *pBAD* promoter were PCR amplified and digested with I-SceI and PI-PspI before being individually ligated to pIB1A1_0, obtaining iBrick plasmids harboring parts of *crtB*, *crtI* and *pBAD* promoter. These plasmids were named as pIB1A1_0-C000002, pIB1A1_0-C000003 and pIB1A1_0-P000001, respectively. Parts were verified through DNA sequencing with primer iPrimer1 and primer iPrimer2.

To assemble the *crt* cluster, part IBP_C000003 (*crtI*) was firstly released from pIB1A1_0-C000003 with I-SceI and PI-PspI digestion and was then cloned into the PI-PspI site of pIB1A1_0-C000002 to obtain pIB1A1_0-X000001, where the head of *crtI* was ligated to the tail of *crtB*. Similarly, *crtE* was cut from pIB1A1_0-C000001 with I-SceI and PI-PspI and cloned into the PI-PspI site of pIB1A1_0-P000001 to obtain pIB1A1_0-X000002, which contains *crtE* and *pBAD*. Finally, *crtBI* genes were cut from pIB1A1_0-X000001 and inserted to the PI-PspI site of pIB1A1_0-X000002, obtaining pIB1A1_0-X000003, which contains the *pBAD*-driven *crt* expression cluster. To avoid self-ligation, the PI-PspI-digested vector should be dephoshorylated, *e.g.* by Antarctic Phosphatase (NEB) in this study.

To test the carotenoid expression, pIB1A1_0-X000003 was transformed into *E. coli* strain DH5α and the obtained strain was cultured in LB medium supplemented with arabinose at a final concentration of 10%, employing DH5α transformed with plasmid pJK5 (**Fig. S1**) as a negative control. After shaking at 37°C for 12 h, cells were harvested and lysed with equal volumes of acetone. Chromatography was performed with Agilent LC 1200/Accurate Mass 6520A QTOF (Agilent Technologies) with a well-plate auto-sampler and the analytical column was Agilent Zorbax XDB-C18 4.6*50 mm, 1.8 µm. The system was operated in isocratic mode with a flow of 0.3 ml/min of 42% ACN mixed with 42% MeOH and 16% dichloromethane. As lycopene is a little, highly symmetric and non-polar molecule, APCI (Atmospheric-pressure chemical ionization) was applied to ionize lycopene.

### Protocol for manipulation and heterologous expression of the actinorhodin biosynthetic cluster (*act*)

Base plasmid pIB2K1_0-C000001 was PCR amplified with primers of iBrick-actF and iBrick-actR to obtain PCR products with homologous sequences of the left and right arms of *act* cluster at both ends. Cosmid N07_85 containing the whole *act* cluster was transformed into *E. coli* strain BW25113 (pIJ790) [32], and the resulted strain was then used to prepare electro-competent cells for transformation of the above PCR products to perform gap repair assay [33]. Transformants were selected on LB plate containing kanamycin, and the obtained candidate plasmids were then verified by both their sizes and their restriction patterns, with correct clones named as pIB2K1_0-K000001. The integration cassette containing the phiC31 integrase and the oriT was firstly PCR amplified from pSET152 [34] and cloned into the I-SceI and PI-PspI sites in pIB1A1_0-C000001, obtaining pIB1A1_0-X000004. Then, the integration cassette was released by I-SceI and PI-PspI digestion and introduced into the same sites of pIB2K1_0-C000001 to obtain pIB2K1_0-X000004. To conjugate a plasmid into *Streptomyces*, *E. coli* strain ET12567 (pUZ8002) [32], which carries the kanamycin and chloramphenicol resistances, is often used. And the kanamycin resistance in pIB2K1_0-X000004 was further replaced with apramycin resistance gene (*apr*) and the obtained plasmid was called pIB2Am1_0-X000004. Finally, the *act* cluster was released from pIB2K1_0-K000001 by I-SceI and PI-PspI digestion and inserted into the PI-PspI site of pIB2Am1_0-X000004 to get pIB2Am1_0-X000005, an iBrick plasmid containing the *act* expression cluster and an integration cassette. Plasmid pIB2Am1_0-X000005 was then conjugated into *Streptomyces* 4F strain for heterologous expression of actinorhodin following the procedures previously described [14].

## Results

### Assembly of *crt* cluster with iBrick parts

**Figure 3:**
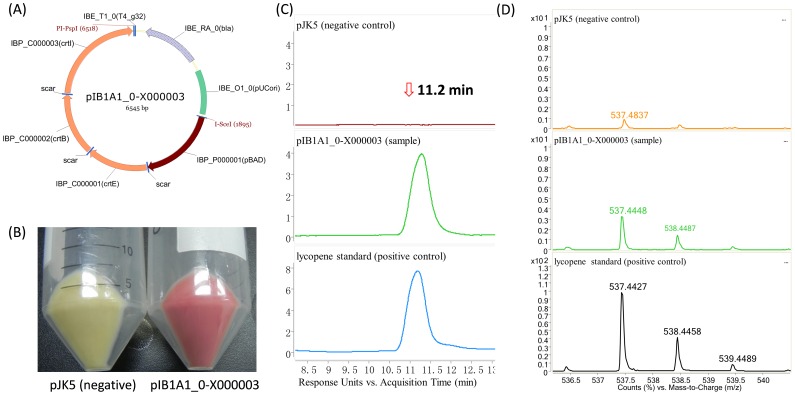
Assembly and expression of the *crt* cluster in *E. coli*. (**A**) Constructed expression plasmid pIB1A1_0-X000003 for *crt* cluster expression. Plasmid map was drawn with Vector NTI Software (Life Technologies). (**B**) Production of carotenoids in *E. coli*. Strain harboring pJK5 was used as a negative control. (C&D) Detection of the product of pIB1A1_0-X000003 with Agilent LC 1200/Accurate Mass 6520A QTOF (Agilent Technologies). (**C**) When analyzed with LC, product of pIB1A1_0-X000003 had the same retention time of 11.2 min as the lycopene standard. No obvious peaks were observed at this retention time in the negative control. (**D**) Mass analysis proved the fermentation product had the same molecular weight as the standard. Tiny peaks were observed in the negative control, which might be caused by contamination. In both C and D, the scales of the diagrams were adjusted to be the same in the tested sample (pIB1A1_0-X000003) and the negative control.

To construct the biosynthetic cluster of carotenoid with the iBrick standard, three genes of *crtEBI* were individually PCR amplified from BioBrick BBa_K274200 and then cloned into an iBrick base vector pIB1A1_0 to obtain pIB1A1_0-C000001 - pIB1A1_0-C000003, respectively. Each part contains a separate ribosome binding site (RBS) and is prefixed with I-SceI and suffixed with PI-PspI. Similarly, the arabinose inducible promoter *pBAD* was prepared as an iBrick part of IBP_P000001. Then, these four parts were assembled together to produce pIB1A1_0-X000003, in which, the carotenoid biosynthetic cluster (*crt*) is expressed under the control of *pBAD* (**Fig. 3A**). Totally, two rounds of hierarchical forward assembly, including three manipulation steps were performed with efficiency no less than 75% in each step (**Table 1**), which should be sufficient enough for most assembly tasks.

**Table 1:** Ligation efficiency of iBrick assembly.

Fragments	Obtained plasmids (kb)	Obtained clones*	Ratio (positive/tested)
**Lycopene**			
Vector+*pBAD-crtE*	pIB1A1_0-X000001 (4)	2264	75% (9/12)
Vector+*crtBI*	pIB1A1_0-X000002 (4.2)	2294	75% (9/12)
Vector+*pBAD-crtEBI*	pIB1A1_0-X000003 (6.5)	1984	75% (186/248)
**Actinorhodin**			
Vector+*act*	pIB1A1_0-X000005 (31)	592	25% (3/12)

* Clones on plates were evenly divided into 4 even sections with only one counted and the total number was then roughly calculated as four times of the number.

The assembled *crt* cluster was then tested in *E. coli* strain DH5α to examine its biological function, employing an isogenic strain but harboring an intermediate plasmid (pJK5, without the *crt* cluster, **Fig. S1**) as a negative control. After arabinose induction, clones with the whole cluster turned red while those without *crt* stayed white (data not shown), and the product of *crt* cluster was further confirmed to be lycopene with LC-MS analysis (**Fig. 3**).

### Cloning and heterologous expression of *act* cluster using iBrick standard

**Figure 4:**
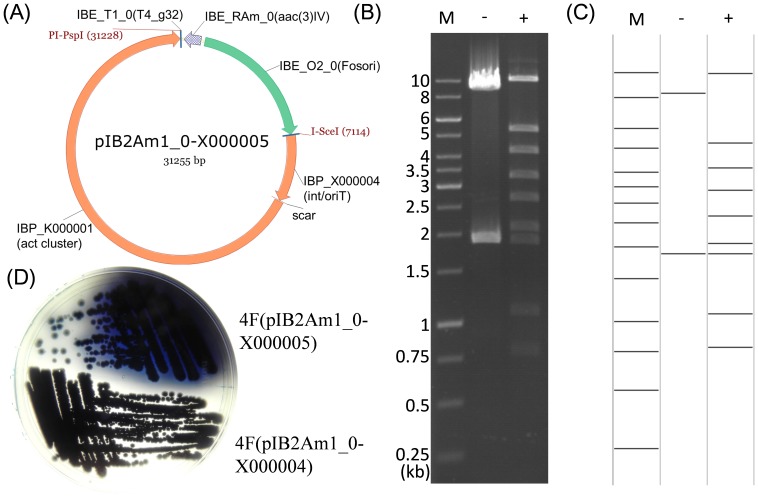
Heterologous expression of the *act* cluster in *Streptomyces* 4F. (A) Constructed expression plasmid pIB2Am1_0-X000005. (B&C) Verification of pIB2Am1_0-X000005 with both the experimental digestion (B) and the electronic restriction (C) with XcmI. M, 1 kb DNA ladder with sizes labeled; -, pIB2Am1_0-X000004; +, pIB2Am1_0-X000005. For “−”, theoretical sizes are 8.32 and 1.88 kb; for “+”, sizes are 9.93, 5.28, 4.18, 3.38, 2.67, 2.08, 1.88, 1.08 and 0.78 kb. Same patterns were obtained for the experimental group and the electronic analysis group. (D) Heterologous expression of *act* cluster in *Streptomyces* 4F, employing pIB2Am1_0-X000004 as a negative control. Strains were cultured on R2YE plate at 30°C for 2 days.

Vector pIB2K1_0, which contains a FOSori and an inducible high copy number origin oriV, is used as the base vector to clone large iBrick parts, *e.g.* the *act* cluster in this study (>20 kb). Gap repair technology [33] was employed to directly clone the *act* cluster into vector pIB2K1_0 to obtain pIB2K1_0-K000001. The phiC31 integrase, *attP* site and the oriT element, which are necessary for conjugation and integration in *Streptomyces*, were together prepared as an iBrick part (IBP_X000004). Part IBP-K000001 containing the *act* cluster was then ligated to the tail of part IBP_X000004, obtaining an iBrick plasmid (pIB2Am1_0-X000005, **Fig. 4A–4C**) for heterologous expression of actinorhodin in a fast growing thermophilic actinomycete, *Streptomyces* 4F [26]. After incubation for three days on R2YE plate, 4F harboring the *act* cluster successfully expressed the blue pigmented actinorhodin, demonstrating the robustness and convenience of iBrick standard in manipulation of large DNA pieces (**Fig. 4D**). Notably, although the ligation efficiency (25%) for construction of pIB2Am1_0-X000005 is remarkably lower than those for small pieces, it should still be sufficient enough for most projects (**Table 1**).

## Discussion

The idea of BioBricks standard was developed to introduce the engineering principles, including standardization, decoupling and abstraction into the field of synthetic biology [10]. Since the birth of the original BioBricks standard (BBF RFC 10), a lot of efforts have been made to improve the standard and enrich the parts library. Participants include scientists around the world, *e.g.* iGEM students, graduate students, postdocs and principal investigators. Till today, at least 103 BBF RFC schemes have been publicly available on the website of BBF and over 20,000 BioBricks parts have been constructed, many of which have been deposited in the iGEM Registry and are widely reused. More importantly, the iGEM Registry not only collects the physical parts from worldwide iGEMers but also provides an open platform for judging the popularity and robustness of each part, which is quite instructive for new users (http://parts.igem.org/Main_Page). With the development of BioBricks, many detailed rules have been gradually set up for the whole system, including the nomenclature, symbols and so on, and a lot of them have been tested in practical applications by different research groups. Therefore, iBrick standard accepts a majority of the present rules and will only make necessary supplements and improvements.

### Description of iBrick standard

Unlike type IIP REs that are commonly used in BioBricks standards, I-SceI and PI-PspI are HEs that produce nonpalindromic ends and therefore two enzymes instead of four are sufficient for orderly assembly of iBrick parts. In addition, as both I-SceI and PI-PspI recognize long DNA sequences (>18 bp), there are rare such sites in natural DNA sequences and therefore basically there is no need for modification of internal DNA sequences (*e.g.* removal of internal restriction sites) during the parts assembly processes.

Among hundreds of thousands of parts to be cloned, components other than protein-coding genes are limited for module or vector constructions, *e.g.* the replication origins, the antibiotic resistance cassettes for selection and the transcriptional terminators, and therefore these components are prepared separately and are prefixed and suffixed with typical type IIP REs in iBrick standard. Rare-cutting REs that recognize 8-bp DNA sequences, including PacI, NotI, SwaI and AscI are used to link these components, which may greatly facilitate the exchange of these components during vector preparation. Specifically, prefixes/suffixes chosen are SwaI/NotI for the replication origins, NotI/PacI for the antibiotic resistance cassettes and AscI/PacI for the transcriptional terminators (**Figs. S1 and S2**). Other DNA sequences, including CDS, promoters, clusters, or pathways are defined as iBrick parts and should be prefixed with I-SceI and suffixed with PI-PspI. In addition, the above mentioned components, including replication origins, antibiotic resistance cassettes and transcriptional terminators can also be prepared as iBrick parts with I-SceI as the prefix and PI-PspI as the postfix as well. However, these components should only be used in construction of vectors with special purposes, *e.g.* vectors containing two or more different replication origins for different hosts or with two or more different antibiotic resistance cassettes for different selection purposes, and in such circumstances, the second component can be added as an iBrick part following the iBrick standard.

The replication origin of the vector must have its capacity in corresponding to the size of the parts being cloned. In this study, the pUCori and a fosmid-derived origin (FOSori) [31] are used in corresponding to the cloning of small *versus* large parts, respectively.

The iBrick standard much resembles other BioBrick standards with respect to the usage of classic restriction and ligation techniques. Definition of iBrick constituents follows that of BioBrick, including parts, vectors and plasmids (**Table 2**). An iBrick part can be prepared through either direct PCR amplification or other techniques to introduce the prefix and suffix at both ends of the part, which is then digested with I-SceI and PI-PspI before being ligated to an iBrick vector with compatible cohesive ends. An iBrick plasmid thus prepared is used to reserve the iBrick part, which should normally be verified either by DNA sequencing or functional analyses prior to be assigned a serial number and stored in the registry. To carry out an assembly, an iBrick part can be firstly released from an iBrick plasmid *via* I-SceI and PI-PspI digestion and then ligated to an iBrick vector, to produce a new iBrick plasmid (**Fig. 1**). The iBrick vector is usually made from an iBrick plasmid with either I-SceI digestion or PI-PspI digestion, depending on the direction of the assembly procedure, *i.e.* PI-PspI digestion allows a forward assembly while I-SceI digestion should be used in the reverse assembly. As only one enzyme cutting is used to prepare the vector, dephosphorylation by alkaline phosphatase is necessary to avoid self-ligation of the vector.

**Table 2:** Letter allocations for parts and elements in iBrick standard.

iBrick Parts (Prefixed with I-SceI and suffixed with PI-PSPI)	
Abbreviations	Categories
IBP_C	CDS
IBP_K	Cluster/Pathway
IBP_P	Promoter
IBP_S	RBS Sequences
IBP_X	Combined parts
IBP_U	Unclassified
IBP_I	BioBrick iGEM parts
IBP_B	BglBrick parts
IBP_O	Replication Origin, built in iBrick standard
IBP_R	Antibiotic Resistance, built in iBrick standard
IBP_T	Transcriptional Terminator, built in iBrick standard
**iBrick Elements (Prefixed/suffixed with type IIP enzymes)**	
Abbreviations	**Categories**
IBE_O	Replication Origin (SwaI/NotI)
IBE_R	Antibiotic Resistance (NotI/PacI)
IBE_T	Transcriptional Terminator (PacI/AscI)

Take the forward assembly for example (**Fig. 2**), the 3′ terminus of part A is ligated to the 5′ terminus of part B to obtain part AB and a 21-bp scar of “5′-GGCAAACAGCTATTATCCCTA-3′” is produced in between. The newly obtained part AB is flanked with I-SceI at the 5′ end and PI-PspI at the 3′ end and therefore partAB can be taken as a new iBrick part for subsequent assembly, allowing iterative assembly of multiple parts. In most cases, the scar is merely a piece of short DNA sequence that links iBrick parts A and B. However, when iBrick standard is used for construction of plasmids to express fusion proteins, the translation stop codon in part A should be omitted and the scar should be translated in frame, which encodes a linker peptide of Gly-Lys-Gln-Leu-Leu-Ser-Leu. Although the scar contains no rare codons when expressed in *E. coli*, the protein linker contains three closely spaced leucines that may cause problems with respect to protein solubility, probably due to the large hydrophobic nature of leucine side chains. Considering the fact that HEs always tolerate a small number of base substitutions within their target DNA sequences without a loss in their enzyme efficiencies [35], [36], one possible solution would be to slightly alter their targets, which would still be recognized and digested by I-SceI and PI-PspI but generate a new scar that encodes a more optimal protein linker for the solubility of the fusion protein.

### Nomenclatures of iBrick standard

To avoid confusion, a nomenclature system is established for iBrick. The iBrick system can be divided into parts (including parts, devices and even systems), vectors and plasmids. For the parts, a specific name is given to each of them as “IBP_$N”, where “IB” stands for iBrick, “P” stands for Parts, an alphabetic letter, represented by “$” indicates the categories of the parts (**Table 2**) and “N” is a unique identification given with a six-figure Arabic number.

Besides “parts”, other components that compose a vector are called “elements”, including replication origins, antibiotic resistance cassettes, transcriptional terminators, *etc*. An *element* is different from a *part* in their prefixes and suffixes and they are distinguished in their nomenclatures, *i.e.* “IBE_$N_n”, where the letter “E” stands for Elements; “$” stands for an alphabetic letter that indicates the specific type of the elements; “N” is a unique identification given with an Arabic number indicating the subcategory of a given type; and “n” indicates the version of the element (**Tables 2** **&** **3**). For example, an inducible FOSori is designated as IBE_O2_0 for an iBrick Element of replication origin (O) –fosmid ori (replication origin category 2) of version 0; and the original kanamycin resistance cassette is given the nomenclature of IBE_RK_0 for an iBrick Element of antibiotic resistance cassette – (kanamycin resistance gene, K) of version 0. Normally, elements are stored as iBrick base-plasmids and can be simply prepared through restriction of the plasmids with type IIP REs followed by gel purification. Based on the nomenclature of elements, vectors are designated as pIB(O)(R)(T)_n, similar to that of BioBrick [8], where “O” indicates the replication origin; “R” indicates the antibiotic resistance (**Table 4**); “T” indicates the type of terminator chosen; and “n” indicates the version of the vector.

**Table 3:** iBrick elements used in this study.

Systematic name	Common name	Version	Type	Source
IBE_O1_0	pUCori	0	replication origin	pUC18 [30]
IBE_O2_0	inducible FOSori	0	replication origin	pCC2FOS (Epicentre)
IBE_RA_0	*bla*	0	ampicillin resistance	pUC18
IBE_RK_0	*kan*	0	kanamycin resistance	pET28a (Novagen)
IBE_RC_0	*cat*	0	chloramphenicol resistance	pCC2FOS
IBE_RAm_0	*aac3(IV)*	0	apramycin resistance	pSET152 [34]
IBE_RH_0	*hyg*	0	hygromycin resistance	pML814 [44]
IBE_T1_0	T4_g32	0	transcriptional terminator	T4 phage [45]

**Table 4:** Letter abbreviations for antibiotic resistance markers in iBrick standard*.

Code	Antibiotics
A	ampicillin
Am	apramycin
C	chloramphenicol
E	erythromycin
G	gentamycin
H	hygromycin
K	kanamycin
N	neomycin
Na	nalidixic acid
R	rifampicin
S	spectinomycin
St	streptomycin
T	tetracycline
Tm	trimethoprim
Ts	thiostrepton
Z	zeocin

* Most of the letter abbreviations are the same as those described by Shetty *et al.* [8].

A plasmid constructed by a vector and a part is given a conjoint name combining the vector and the part, *e.g.* part IBP_C000001 ligated to vector pIB1K1_0 results in a plasmid pIB1K1_0-C000001 (**Table 5**). When multiple parts are assembled together, a new serial number must be given to indicate the combination of these parts. Besides, the same parts with altered arrangements must be assigned with a different identification number.

**Table 5:** iBrick parts used in this study.

Systematic name	Common name	Vector	Type	Source
IBP_C000001	*crtE*	pIB1A1_0	CDS	BioBrick BBa_K274200
IBP_C000002	*crtB*	pIB1A1_0	CDS	BioBrick BBa_K274200
IBP_C000003	*crtI*	pIB1A1_0	CDS	BioBrick BBa_K274200
IBP_K000001	*act* cluster	pIB2K1_0	Cluster	fosmid N07_85 (this lab)
IBP_P000001	*pBAD*	pIB1A1_0	Promoter	pKD46 [43]
IBP_X000001	*crtBI*	pIB1A1_0	Combined	IBP_C000002, IBP_C000003
IBP_X000002	*pBAD-crtE*	pIB1A1_0	Combined	IBP_C000001, IBP_P000001
IBP_X000003	*pBAD-crtEBI*	pIB1A1_0	Combined	IBP_P000001, IBP_C000001-3
IBP_X000004	phiC31 int, *oriT*, *attP*	pIB1A1_0/pIB2K1_0/pIB2Am1_0	Combined	pSET152 [34]
IBP_X000005	phiC31 int, *oriT*, *attP*, *act* cluster	pIB2Am1_0	Combined	IBP_X000004, IBP_K000001

### Possible applications of iBrick in future

The DNA sequences recognized by homing nucleases used in iBrick are extremely rare in natural DNA sequences, bringing much convenience in preparation of iBrick parts, especially for those large DNA pieces. In this study, the entire *act* biosynthetic cluster was directly taken from a cosmid with gap repair and stored as an iBrick part. Using iBrick assembly, this large part can be easily operated, *e.g.* ligated with an integrating cassette for heterologous expression. Because many secondary metabolites biosynthetic clusters are mined and produced in either its original host or a phylogenetically close host, there is usually no need to perform codon optimization. For example, polyketide biosynthetic clusters from actinomycetes are preferably characterized and optimized for industrial production in an actinomycete host rather than in an *E. coli* strain. With the availability of a huge number of bacterial genomic data (including both finished and unfinished), the number of predicted clusters for secondary metabolites grows rapidly. However, a lot of these clusters are cryptic and promoter engineering might be necessary for further characterization. Simply, an inducible strong promoter can be added in front of the whole cluster to drive its expression, instead of substitution of its original promoter. Therefore, the whole cluster can actually be cloned as a large iBrick part and then ligated with another part of an inducible promoter, using iBrick assembly. Moreover, with the development of one-step cloning techniques of large DNA pieces, *e.g.* TAR [37] and RecE-mediated LLHR [38], large iBrick parts can also be cloned directly from genomic DNAs through combination of these efficient cloning methods. Besides, for cloning of those extremely large DNA pieces (>100 kb), which might be too large to be directly cloned, iBrick standard might be effective in combination with other assembly techniques, *e.g.* the Site-Specific Recombination based Tandem Assembly method (SSRTA) [39]. SSRTA uses the phiBT1 integrase mediated efficient recombination between paired mutated *attB* and *attP* sites [40]. Therefore, a large part can firstly be divided into several subparts, which can be directly cloned with one-step strategies; then, adaptors containing paired *attB* and *attP* sites can be ligated to the subparts that are released from I-SceI and PI-PspI digestion and SSRTA can subsequently be employed to accomplish the assembly of the large part.

Considering the large size of some parts, a combined origin with ori2 and oriV [31] is used in iBrick standard. When the plasmid is produced in a strain containing the *araC–P*_BAD_ driven *trfA* gene, the copy number can be induced from single copy to 10–200 copies per cell, simply through addition of arabinose [31]. Because many parts may express toxic products that are harmful to the hosts, control of both the copy number of the plasmid and the promoter activities are extremely useful for cloning and preservation of the toxic parts without killing the hosts. When the plasmids need to be prepared in a large population, inducers can be added when the cell density reaches high and cells can be harvested for plasmids preparation in a short time. Besides, the induction of the copy number of plasmids containing oriV can also be achieved through co-transformation of pJK46 (**Fig. S3**), which expresses TrfA in any *E. coli* strains.

### Developments of iBrick in future

Due to the long recognition sequences, the 21-bp scar produced in iBrick is much longer than routine BioBricks standards (*e.g.* 6 bps in BglBricks [9]) and iBrick is therefore more suitable in assembling large parts instead of small parts (*e.g.* transcriptional terminators and RBSs). During the process of construction and storage of iBrick plasmids that contain scars, no recombination between these scars has been observed in *E. coli* hosts. Besides, the constructed plasmid containing scars seems quite stable in Streptomyces 4F. Taken together, although the *in vivo* recombination between scars cannot be totally expelled, the efficiency should be extremely low. Besides, the recombination efficiency in other hosts, including *Saccharomyces* and cell lines, is still unclear at the moment and needs be investigated in future studies.

A non-cleaving mutant of I-SceI was ever successfully linked to FokI, a type IIS RE, generating a new type of IIS REs of CdnDs [41]. Interestingly, CdnDs recognize long DNA sequences just as I-SceI but cut DNA outside of their recognition sites, thus promising in application of scar-less assembly of large DNA pieces. To develop a scar-less assembly scheme for iBrick standard, PI-PspI needs to be engineered in a similar way to acquire a hybrid type IIS RE that recognizes PI-PspI recognition sequences but cuts DNA outside.

Because different enzymes are used in iBrick standard and BioBrick RFC 10 (the most popular BioBricks standard), the two standards are not compatible with each other at the moment. Considering a large number of parts have been collected by BioBrick Registry using the RFC 10 standard, this incompatibility needs to be solved by persistent developments of iBrick standard in future.

The optimal reaction temperature for PI-PspI is 65°C and cannot be inactivated by heating. Therefore, to make a double digestion, I-SceI is firstly added and the reaction is performed at 37°C in PI-PspI buffer, then PI-PspI is added directly and the digestion is transferred to 65°C. Besides, PI-PspI seems extremely stable, and the procedure combining gel electrophoresis and column purification is preferable for removal of PI-PspI than other treatments, *e.g.* SDS, proteinase K or trypsin (data not shown). Therefore, efforts are now making to engineer PI-PspI to lower its optimal reaction temperature and make it easier to be inactivated, *e.g.* by heat inactivation. Moreover, to better support iBrick standard, establishment of a bioinformatics platform as well as development of useful tools are now under way.

## Supporting Information

Figure S1 **Plasmid map of pJK5.** The construction procedures can be found in *Materials and Methods*. Plasmid pJK5 was used as a negative control in analysis of carotenoid production.
(TIF)

Figure S2 **Plasmid map of pIB1A1_0-C000001.** Plasmid pIB1A1_0-C000001 is used as a base plasmid for construction of iBrick parts. Base vector pIB1A1_0 can be prepared *via* I-SceI and PI-pspI double digestion of the base plasmid, followed by gel electrophoresis and gel purification.
(TIF)

Figure S3 **Plasmid map of pJK46.** The *trfA* gene was firstly amplified from pRK415 [42] with primers of trfA-F2 and trfA-R4 and the amplicon was then inserted into the HincII site of pUC18. After being verified by DNA sequencing, *trfA* gene was then released from pUC18 through EcoRI and SmaI digestion, and introduced to the same sites of pKD46 [43] to obtain pJK46.
(TIF)

Figure S4 **Frequently used base vectors.** The construction processes can be found in *Materials and Methods*. Base vectors are used for preparation of iBrick parts and subsequent assembly.
(TIF)

Table S1 **Primers used in this study.**
(DOCX)
