# Supplementary figures and images for: iBrick: A New Standard for Iterative Assembly of Biological Parts with Homing Endonucleases
Source: PLoS One. 2014 Oct 20;9(10):e110852. doi: 10.1371/journal.pone.0110852 (PMC4203835; doi:10.1371/journal.pone.0110852)

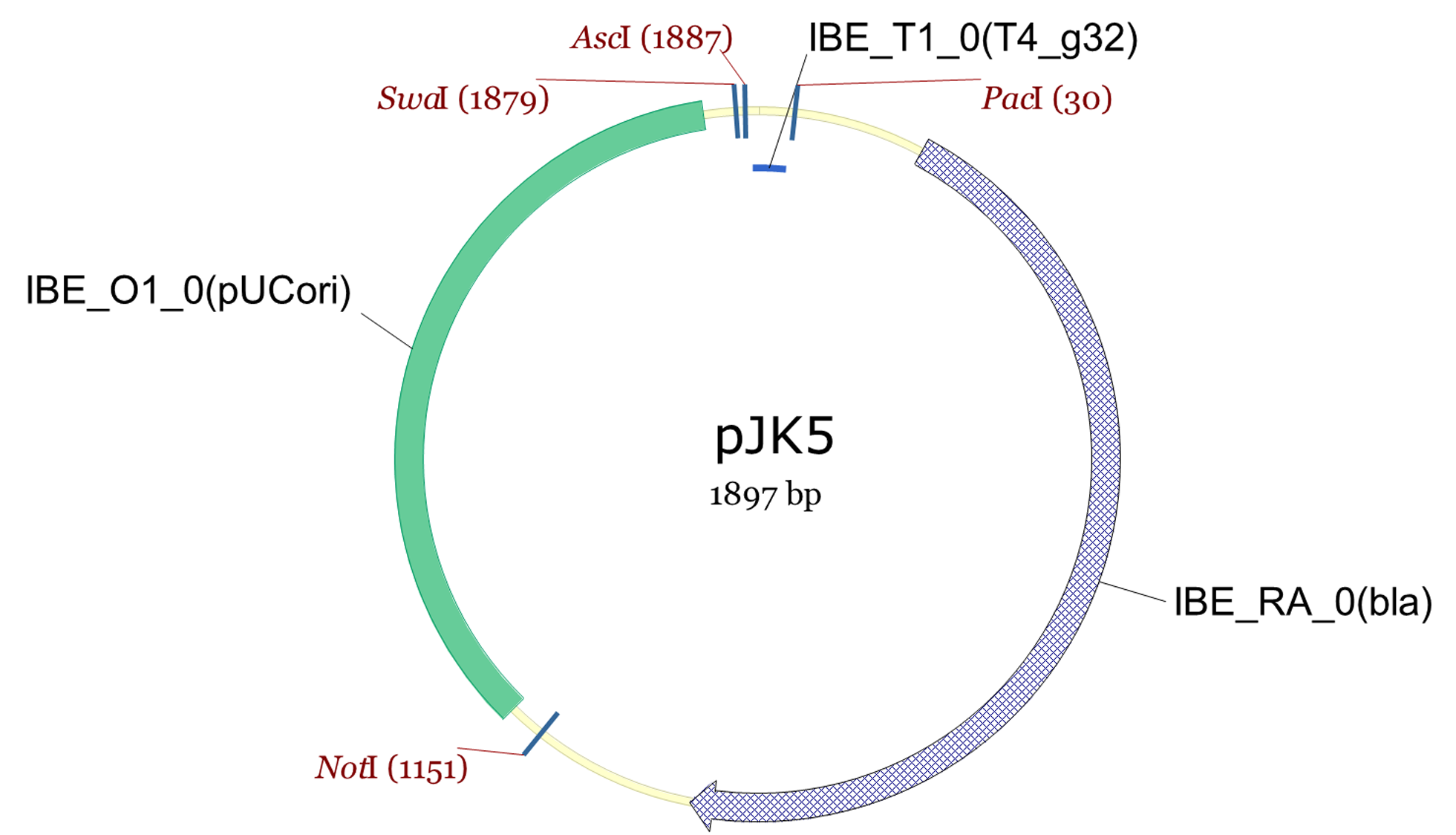

Supplement: Figure S1 — Plasmid map of pJK5. The construction procedures can be found in Materials and Methods. Plasmid pJK5 was used as a negative control in analysis of carotenoid production. (TIF) [file pone.0110852.s001.tif]

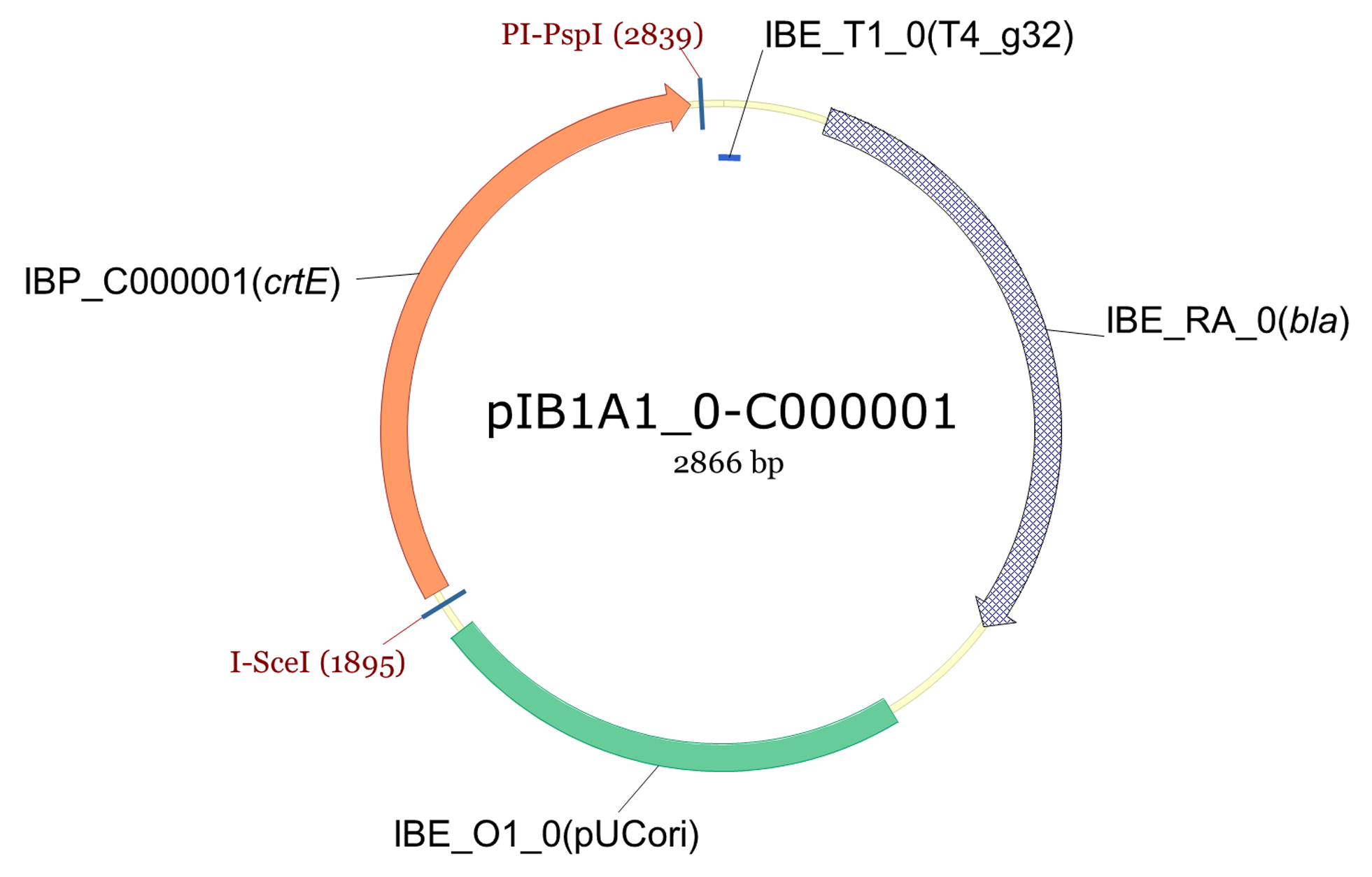

Supplement: Figure S2 — Plasmid map of pIB1A1_0-C000001. Plasmid pIB1A1_0-C000001 is used as a base plasmid for construction of iBrick parts. Base vector pIB1A1_0 can be prepared via I-SceI and PI-pspI double digestion of the base plasmid, followed by gel electrophoresis and gel purification. (TIF) [file pone.0110852.s002.tif]

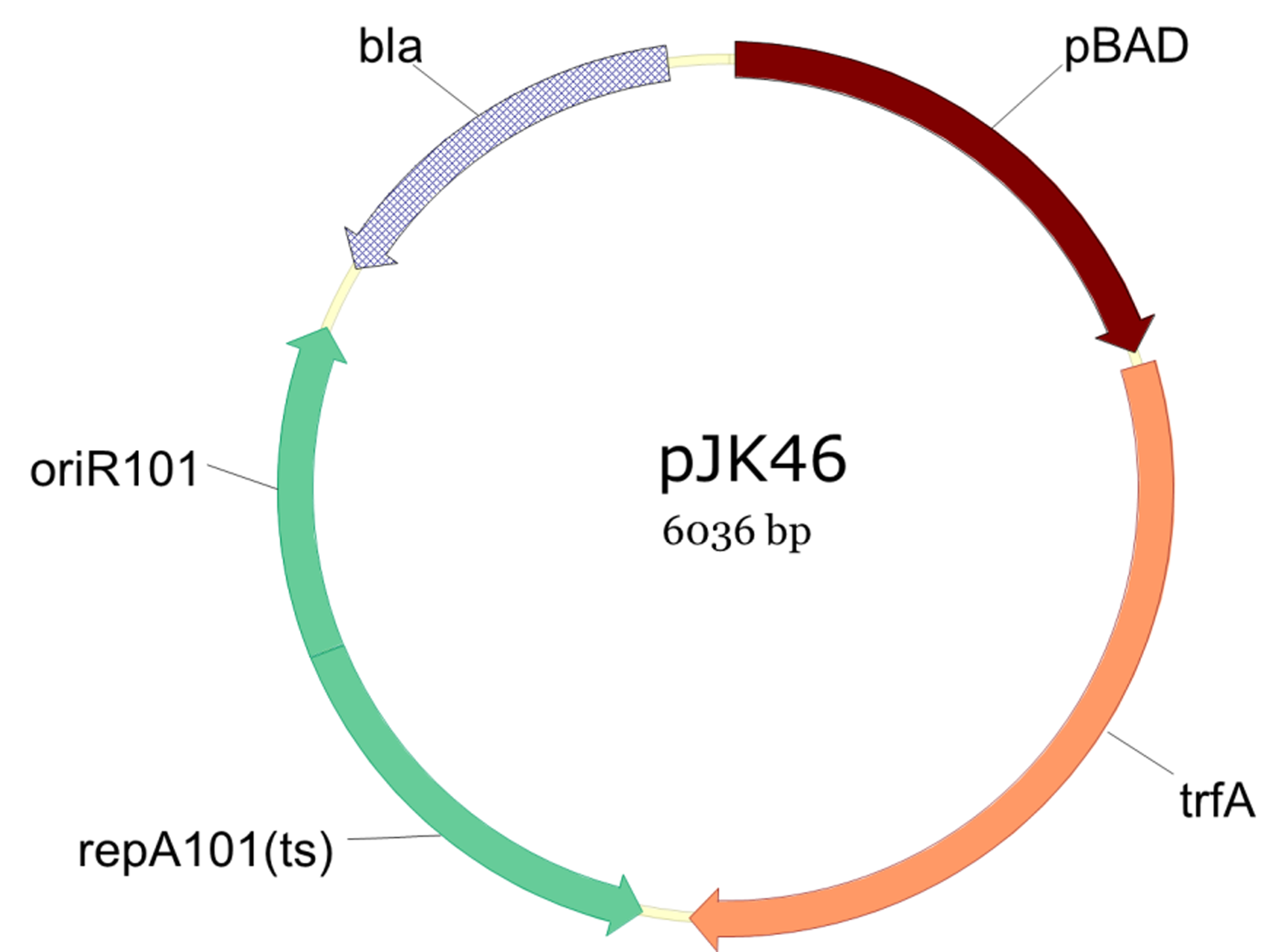

Supplement: Figure S3 — Plasmid map of pJK46. The trfA gene was firstly amplified from pRK415 [42] with primers of trfA-F2 and trfA-R4 and the amplicon was then inserted into the HincII site of pUC18. After being verified by DNA sequencing, trfA gene was then released from pUC18 through EcoRI and SmaI digestion, and introduced to the same sites of pKD46 [43] to obtain pJK46. (TIF) [file pone.0110852.s003.tif]

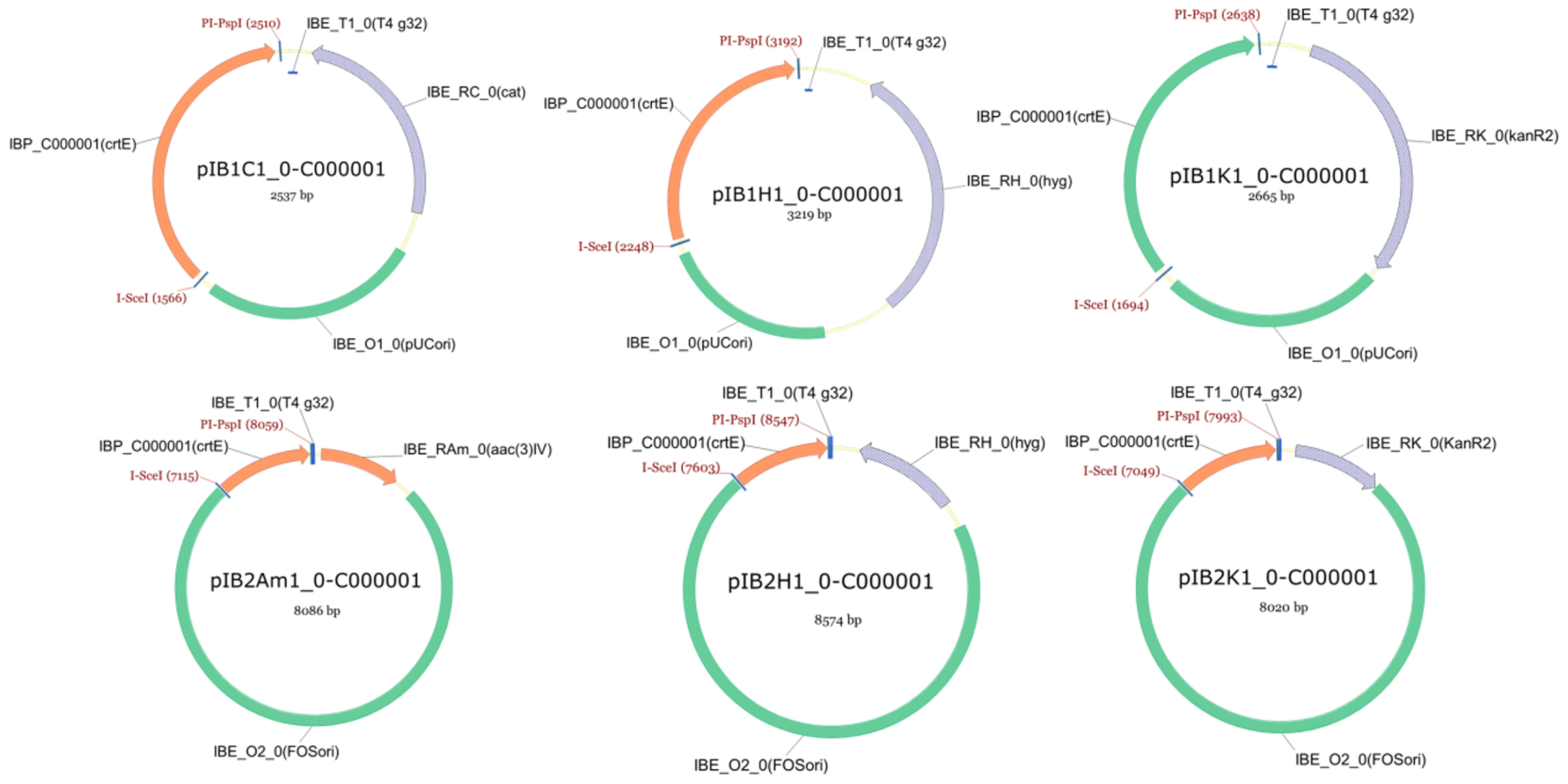

Supplement: Figure S4 — Frequently used base vectors. The construction processes can be found in Materials and Methods. Base vectors are used for preparation of iBrick parts and subsequent assembly. (TIF) [file pone.0110852.s004.tif]
