# Supplementary material for: iBrick: A New Standard for Iterative Assembly of Biological Parts with Homing Endonucleases
Source: PLoS One. 2014 Oct 20;9(10):e110852. doi: 10.1371/journal.pone.0110852 (PMC4203835; doi:10.1371/journal.pone.0110852)
Supplement: Table S1 — Primers used in this study. (DOCX) [file pone.0110852.s005.docx]

**Supplementary Data**

**Table S1. Primers used in this study**

| Name | Sequences of primers | Product name | Additional information |
| --- | --- | --- | --- |
| **For construction of base vectors** | | | |
| iBrick-Ori(pUC)-1 | aaataagcggccgcaggggataacgcaggaaaga | Replication origin | pUC18 [30] |
| iBrick-Ori(pUC)-2 | tccccggcgcgccttatttaaatttcgttccactgagcgtca |  |  |
| iBrick-Ori(FOS)-1 | aaataagcggccgctttagcttccttagctcctg | Replication origin | pCC2FOS (Epicentre) |
| iBrick-Ori(FOS)-2 | gggggcatttaaatggtaaccaggtattttgtcc |  |  |
| iBrick-AP1 | ttaattaagaaatgtgcgcggaaccccta | Ampicillin resistance gene | pUC18 |
| iBrick-AP2 | gcggccgcctgacgctcagtggaacgaa |  |  |
| i-Brick-Kan-F | aaataagcggccgcggggtctgacgctcagtggaac | kanamycin resistance gene | pET28a (Novagen) |
| i-Brick-Kan-R | cccccgcttaattaacttagaaaaactcatcgagcatc |  |  |
| i-Brick-Cm-F | aaataagcggccgcgtaagaggttccaactttcacc | Chloromycin resistance gene | pCC2FOS |
| i-Brick-Cm-R | ccccgcttaattaagcgtttaagggcaccaataactg |  |  |
| i-Brick-Apr-F | aaataagcggccgcacgttaagggattttggtcatg | Apramycin resistance gene | pSET152 [34] |
| i-Brick-Apr-R | ccccgcttaattaacgtcatctcgttctccgctcatg |  |  |
| i-Brick-Hyg-F | aaataagcggccgcttcgttaacgcatgaaatcaatc | Hygromycin resistance gene | pML814 [44] |
| i-Brick-Hyg-R | ccccgcttaattaacggcggctcagcgacgggctcag |  |  |
| **For preparation of iBrick parts** | | | |
| ibrick-pbad-f | ccgattattaccctgttatccctattatgacaacttgac | Arabinose induced promoter | pKD46 [43] |
| ibrick-pbad-r | ccacccacccataatacccataatagctgtttgcccagtagagagttgcg |  |  |
| ibrick-crtE-f | ccgattatttaaatattaccctgttatccctaaaggaggtactagatgacggtctgc | First gene of lycopene biosynthesis | BBa_274100 |
| ibrick-crtE-r | ccacccggcgcgccacccataatacccataatagctgtttgccttaactgacggcagcga | First gene of lycopene biosynthesis | BBa_274100 |
| ibrick-crtB-f | ccgattattaccctgttatccctaaaggaggtactagatgaataatccg | second gene of lycopene biosynthesis | BBa_274100 |
| ibrick-crtB-r | ccacccacccataatacccataatagctgtttgccctagagcgggcgctgcca | second gene of lycopene biosynthesis | BBa_274100 |
| ibrick-crtI-f | ccgattattaccctgttatccctaaaggaggtactagatgaaaccaact acggtaattgg | third gene of lycopene biosynthesis | BBa_274100 |
| ibrick-crtI-r | ccacccacccataatacccataatagctgtttgcctcatatcagatcctccagc | third gene of lycopene biosynthesis | BBa_274100 |
| ibrick-int-f | ccgattattaccctgttatccctaatcggtcttgccttgctc | φBT1 integrase coding gene and oriT | pSET152 |
| ibrick-int-r | ccacccacccataatacccataatagctgtttgccacccgcaggacatatccac | φBT1 integrase coding gene and oriT | pSET152 |
| ibrick-ptet-f | ccgattattaccctgttatccctatacatggctctgctgtagtgag | tetracycline induced promotor | pRK415 [42] |
| ibrick-ptet-r | ccacccacccataatacccataatagctgtttgccttcatgtctggcctccggaccag |  |  |
| ibrick-pLac-f | ccgattattaccctgttatccctaccgagcgcagcgagtcagtgag | IPTG induced promotor | pCA24N [46] |
| ibrick-pLac-r | ccacccacccataatacccataatagctgtttgccagctgtttcctgtgtgaaattg |  |  |
| ibrick-act-f | agcggcccgtggcaccgtcgatcagctcgcggaggatgtcggcgtggccgctgccgtcagttaaggcaaacagc | Actinohordin biosynthetic gene cluster | pIB2K1_0-C000001 |
| ibrick-act-r | ggtcctgtcgttctacggccaccagatgcagaagctggacggccgtgactaccgtcatctagtacctcctttagg |  |  |
| trfA-F2 | ggtggaattcgagctctaaggaggttataaaaaATGAATCGGACGTTTGACCGGAAGGC | *trfA* gene | pRK415 [42] |
| trfA-R4 | cctcCCCGGGATCGTATCGGGCTACCTAGCAGAGC |  |  |
| **Primers for DNA sequencing** | | | |
| iPrimer1 | GCAAGCAGCAGATTACGCGCAG |  |  |
| iPrimer2 | CGCTGTTGAGATCCAGTTC |  |  |

**REFERENCES**

46. Kitagawa M, Ara T, Arifuzzaman M, Ioka-Nakamichi T, Inamoto E, et al. (2005) Complete set of ORF clones of *Escherichia coli* ASKA library (a complete set of *E. coli* K-12 ORF archive): unique resources for biological research. DNA Res 12: 291-299.
